# Air leak phenotyping by mandibular jaw movement analysis in CPAP therapy: Key insights for practitioners

**DOI:** 10.1002/rcr2.70030

**Published:** 2024-09-24

**Authors:** Jean‐Benoit Martinot, Lorent Hostaux, Atul Malhotra, Dennis Hwang, Jean‐Louis Pépin

**Affiliations:** ^1^ Sleep Laboratory CHU Université catholique de Louvain (UCL) Namur Site Sainte‐Elisabeth Namur Belgium; ^2^ Institute of Experimental and Clinical Research UCL Bruxelles Woluwe Brussels Belgium; ^3^ Unité de recherche clinique CHU Université catholique de Louvain (UCL) Namur Site Godinne Yvoir Belgium; ^4^ Division of Pulmonary, Critical Care, and Sleep Medicine University of California San Diego La Jolla California USA; ^5^ Kaiser Permanente San Bernardino County Sleep Center Kaiser Permanente Southern California Fontana California USA; ^6^ University Grenoble Alpes HP2 Laboratory Grenoble France; ^7^ EFCR Laboratory Grenoble Alpes University Hospital Grenoble France

**Keywords:** air leak, CPAP, home sleep testing, mandibular jaw movements, sleep apnea

## Abstract

Monitoring unintentional air leaks in continuous positive airway pressure (CPAP) treatment for obstructive sleep apnea (OSA) is essential for therapy success. While leaks are often attributed to improperly sealed masks, mouth openings may also cause them, requiring interventions. Recent studies demonstrated distinctive mandibular jaw movement (MJM) signal patterns during sleep related to respiratory events and sleep stages. Analysing MJM during CPAP treatment reveals air leak peaks coinciding with maximal MJM amplitude during obstructive events, and air leak decreases corresponding to arousals. Examining leaks with MJM offers valuable insights into their origins and might open new avenues for CPAP management.

## Graphical abstract

Monitoring unintentional air leaks in continuous positive airway pressure (CPAP) treatment for obstructive sleep apnea (OSA) is essential for therapy success. Mandibular jaw movemement signals could provide valuable insights into the origins of air leaks and might open new avenues for CPAP management.

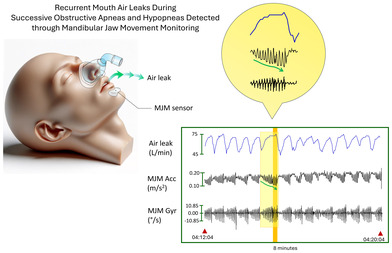

## INTRODUCTION

While continuous positive airway pressure (CPAP) therapy demonstrates high efficacy in enhancing quality of life and reducing daytime sleepiness in obstructive sleep apnea (OSA), long term adherence is a major concern.^1^ CPAP adherence emerges indeed as a critical factor in mitigating symptoms, health resource utilization, and cerebrovascular/cardiovascular risks associated with OSA.^2^ Factors such as disease severity, comorbidities, and the management of CPAP‐related side effects are predictors of CPAP adherence and termination.^1^

Unintentional air leaks during CPAP treatment in OSA patients are commonly observed, occurring at the nasal/oronasal interface when optimal mask sealing is compromised. Such leaks can result from changes in head position or intermittent mask displacement during sleep.^3^ They can lead to significant disruptions in sleep quality and may cause noisy disturbances and conjunctival irritation due to airflow directed towards the eyes. Even with a properly sealed mask, unintentional mouth leaks can occur, potentially causing mouth dryness, and audible breathing. In addition, high outflow air leaks might compromise the efficacy of targeted pharyngeal pneumatic pressurization, contributing to elevated residual apnea/hypopnea indices (AHI) or affecting the accuracy of CPAP machine software in estimating the residual AHI.^4^ All these issues can reduce CPAP adherence and increase the risk of treatment discontinuation.^5^, ^6^ Recognizing these potential complications is essential for comprehensive CPAP therapy management, ensuring proactive measures are in place to address these challenges and improve patient compliance.

Recent studies have revealed distinctive patterns in mandibular jaw movements (MJM) during sleep, associated with various breathing disturbances and sleep stages.^7^, ^8^, ^9^ Basically, the amplitude of MJM informs about the level of respiratory effort (RE) or the muscular engagement needed to ventilate by the diaphragmatic pump and leveraging mandibular activity for strengthening the pharyngeal walls just before diaphragm contraction. A typical obstructive event on MJM recording is characterized by an initial increase in respiratory drive, evidenced by a rise in peak‐to‐peak amplitude, matching respiratory frequency and at the end of the episode by a brisk movement closing the mouth on arousal. Visual data explorations indicate a clear contrast of arousals, which are associated with brisk MJM disrupting the previous sleep period under increased RE. Periods of obstructive sleep‐disordered breathing, recognized as obstructive hypopneas/apneas or respiratory effort‐related arousals (RERAs), coincide with varying degrees of mouth openings, which may fluctuate with each respiratory cycle, potentially leading to air leaks.^9^

This report explores distinctive characteristics of air leak waveforms, highlighting challenges when optimal nasal mask sealing is hindered, compared to mouth air leaks resulting from mouth opening.

## CASE REPORTS

We conducted a comprehensive examination of air leak curves using the ResScan software (ResMed Inc., San Diego, CA, USA) and compared them with raw MJM data captured concurrently by the accelerometer and gyroscope within the sensor (Sunrise, Namur, Belgium) in two OSA patients undergoing CPAP treatment. The OSA diagnosis was established through in‐laboratory polysomnography and both patients were equipped with the AirSense 10 device (ResMed Inc., San Diego, CA, USA) with Mirage FX nasal mask. Air leaks were identified during annual control and meticulously examined. Patients were requested to continue their CPAP treatment and consented to concomitant MJM monitoring at home. Air leak data was subsequently extracted from the CPAP device and transferred to an EDF browser for concurrent visualization and analysis, synchronized with simultaneously recorded MJM data following alignment of the CPAP and Sunrise device clocks. This study was conducted at CHU UCL Namur (site Ste‐Elisabeth, Namur, Belgium). Both patients provided consent for participation in the observational study.

One year after initiating CPAP treatment, patients 1 and 2 were referred to the sleep specialist for OSA reassessment following a persistently high residual AHI and severe mouth dryness, respectively.

**FIGURE 1:**
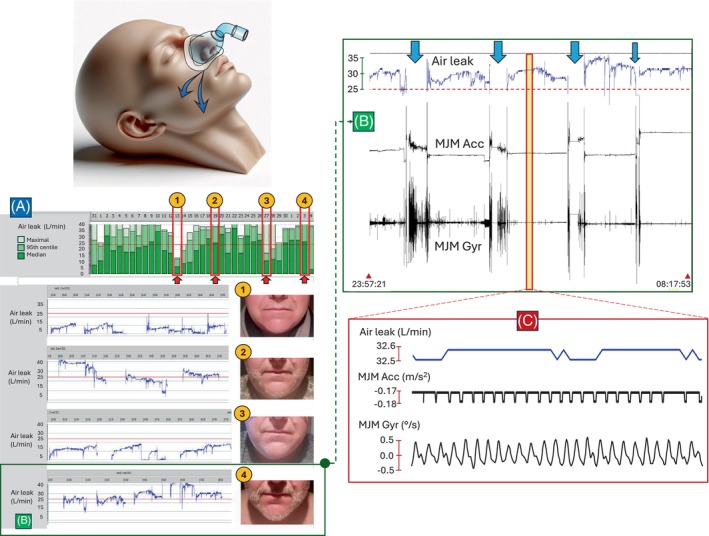
Unintentional air leaks related to facial hair during nasal CPAP therapy. Left panel: (A) The bar graphs depict the air leak outflow in litres per minute (L/min) per day of CPAP usage, categorized by maximal values, the 95th percentile, and the median, as denoted by lightest, light, and dark green colours, respectively. Lower part: The air leak waveform curves, corresponding to four nights (indicated by red arrows and circle labels 1–4). Notably, the red horizontal line signifies an air leak outflow of 24 L/min. Right panel: The air leak waveform curve recorded during the fourth night (B) is displayed alongside simultaneous traces of mandibular jaw movements (MJM) captured by the accelerometer (MJM Acc) and gyroscope (MJM Gyr) integrated into the recording sensor. Data is presented at different levels: (B) a night overview, with blue arrows depicting interruptions in the air leak traces that occur when the patient temporarily removes the mask (such as during visits to the toilet or kitchen for meals), and (C) a zoomed‐in level focusing on a continuous and stable mask air leak during normal sleep. During this period, the gyroscopic rotation aligning with the breathing frequency exhibits minimal angular speed, approximately 1°/s, while the accelerometric displacement is also minimal and stable over time, showing that the mouth is either closed or nearly closed.

***Case 1***: a 56‐year‐old male (BMI = 42.4 kg/m^2^) using a CPAP at a titrated pressure of 10.8 cmH_2_O, improving his AHI from 38.2 to 9.3 events/h. Figure 1A shows leak curves from four nights, with synchronous presentation of air leaks and MJM recorded during the fourth night (Figure 1B,C).

Air leaks are noticeably reduced when the patient is clean‐shaven (nights 1 and 3) compared to previous condition with beard (nights 2 and 4). The highest unintentional air leaks occur with thickest beard, with the air leak outflow plateau reaching 45 L/min during the fourth night. Air leak traces are disrupted by voluntary CPAP pauses (while the removal of the mask is synchronized with the turbine shutdown) during wakefulness, when MJM are irregular and unpredictable (Figure 1B).

In undisturbed sleep, the mandibular jaw undergoes minor physiological shifts of a few tenths of a millimetre around a stable position, keeping the mouth almost closed. These subtle rotational movements, associated with the respiratory cycle and controlled by respiratory centers, are initiated by the rotation of the mandibular condyle within the temporomandibular joint and are well depicted by the gyroscope MJM signal (zoomed‐in part in Figure 1C). Concurrent recording of MJM alongside continuous beard‐related leaks confirms that the patient is genuinely asleep and experiences no respiratory events.

**FIGURE 2:**
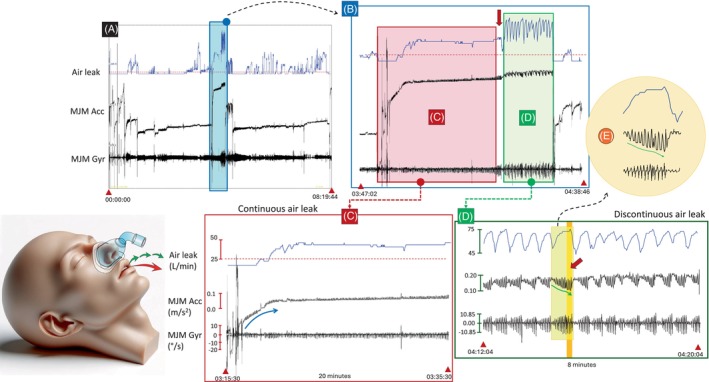
Unintentional air leaks during nasal CPAP therapy in the absence of a beard and moustache. The figure represents typical patterns of air leak waveforms and simultaneous traces of mandibular jaw movements (MJM) captured by the accelerometer (MJM Acc, in m/s^2^) and gyroscope (MJM Gyr, in°/s) during continuous and discontinuous air leaks. Data is presented at four different levels: (A) A night overview of the air leak flow and concomitant MJM signals. The red horizontal line indicates an air leak outflow of 24 L/min. (B) A first zoomed‐in level focusing on a period with continuous and discontinuous mouth air leakages. The red arrow indicates the arousal at the end of the prolonged period of respiratory effort‐related arousal (RERA). (C) A zoomed‐in segment within period (B), focusing on the continuous leakage. The blue arrow signifies a progressive increase in mouth opening. (D) A zoomed‐in segment within period (B), focusing on the discontinuous leakage. The red arrow indicates an arousal at the end of an obstructive apnea or hypopnea. The green arrow indicates a gradual increase in mouth opening synchronized with breathing frequency, as shown in the circle (E). Mouth opening intensifies, as depicted by more and more negative accelerometric signals, until arousal prompts closure to prevent air leakage. Notably, air leakage rises during periods of respiratory effort and abruptly decreases upon arousal. In (D), the MJM signal measures the rotational movements and relative position of the mandible, rather than an absolute change in mouth opening distance.

***Case 2***: a 60‐year‐old female patient (BMI = 39.9 kg/m^2^) used a CPAP with a Mirage FX nasal mask at 11.6 cmH_2_O pressure, improving her AHI from 86.3 to 15.7 events/h. Figure 2B–D illustrate distinct air leak patterns and concurrent MJM signals during continuous and discontinuous mouth air leaks unrelated to facial hair or other factors causing mask unsealing. The continuous leakage shows a baseline shift (Figure 2C), while sharp peaks with brief and abrupt changes in amplitude represent discontinuous mouth air leaks (Figure 2D).

Continuous leakage (Figure 2C) coincides with mouth opening depicted by a high MJM amplitude on the accelerometer signal. This is typically observed during RERAs, where the arousal prompts mouth closure to halt air leak.

Discontinuous leaks (Figure 2D) are associated with repeated obstructive apneas or hypopneas and mouth opening, as indicated by a more negative level on the accelerometer signal, while RE increases (increased MJM amplitude on the gyroscope signal). The arousal (red arrow in Figure 2D) terminates the obstructive event, reducing the air leak. This interplay between air leaks and MJM provides valuable insights into the dynamics of respiratory events during CPAP.

## DISCUSSION

The analysis of MJM concomitantly recorded during CPAP therapy showed that maximal MJM amplitude coincides with peak outflow of air leaks during an obstructive breathing episode, correlating with heightened respiratory effort. Arousals, well depicted by the accelerometer MJM signal showing brisk and abrupt jaw movements indicating mouth closure, correspond to a decrease in air leaks.

The leak measurements by the CPAP device, particularly at high levels, may be subject to potential inaccuracies, as noted by the CPAP manufacturer. While this could impact the absolute values of unintentional leaks, the relative changes in these leaks, as detected through the MJM signal, remain interpretable.

Unintentional air leak waveforms, visualized using CPAP software, reveal information about their continuous or discontinuous nature. Continuous air leaks manifest as a quite stable increase in leakage amplitude (Figures 1B and 2C). However, unlike continuous leaks, discontinuous air leakage exhibits abrupt changes in leak amplitude (Figure 2D).

In absence of residual obstructive episodes, as indicated by regular MJM signals oscillating at the breathing frequency, continuous air leaks can still happen. The occurrence and strength of these unintentional leaks are influenced by factors such as facial hair thickness (Figure 1B). Prolonged residual RERAs during CPAP treatment, as highlighted by MJM traces and possibly due to some degree of nasal obstruction,^10^, ^11^ are also associated to mouth air leaks. These leaks exhibit a characteristic progressive and gradual increase in MJM amplitude corresponding to mouth opening, before reaching a plateau where the level of RE remains stable or slightly increases until arousal‐induced mouth closure stops the leak (Figure 2C). In contrast, discontinuous air leakage, showing abrupt variations in leak amplitude, emerges during obstructive episodes well depicted by the MJM signals, modulated by the residual RE and intensified mouth opening in conjunction with the underlying respiratory drive (Figure 2D). When analysed under CPAP, by reflecting the level of underlying RE, specific MJM patterns provide crucial insights to identify residual obstructive events associated with mouth opening responsible for discontinuous leakage.

In conclusion, MJM analysis, in conjunction with the monitoring of unintentional air leaks in OSA patients during CPAP therapy, provides a thorough assessment of leakage origins. MJM analysis under CPAP aids in identifying sleep/wake states, RERAs, and residual obstructive events linked to different air leak types. Such combined analysis of leak profiles and MJM could therefore significantly enhance patient adherence and CPAP treatment outcomes.

## CONFLICT OF INTEREST STATEMENT

Dr. Martinot reported being a scientific advisor to Sunrise and being an investigator in pharmacy trials for Jazz Pharmaceuticals and Theranexus. Dr. Pépin reported being a scientific advisor to Sunrise; receiving grants and/or personal fees from ResMed, Philips, Fisher & Paykel, Sefam, AstraZeneca, AGIR à dom, Elevie, VitalAire, Boehringer Ingelheim, Jazz Pharmaceuticals, and Itamar Medical Ltd.; and receiving research support for clinical studies from Mutualia and Air Liquide Foundation. He is supported by the French National Research Agency in the framework of the “Investissements d'avenir” program (ANR‐15‐IDEX‐02) and the ‘e‐health and integrated care and trajectories medicine and MIAI artificial intelligence’ chairs of excellence from the Grenoble Alpes University Foundation. This work has been partially supported by MIAI @ Grenoble Alpes (ANR‐19‐P3IA‐0003). Dr. Malhotra is funded by the NIH. He reports income from Jazz, Zoll, Sunrise, Livanova, Eli Lilly related to medical education. ResMed provided a philanthropic donation to UCSD. Dr. Hwang is funded by the NIH and the Kaiser Permanente Clinical Investigator Program. There are no other disclosures to report in relation to this work. This research did not receive any funding or other financial support from Sunrise company. The funding sources had no role in the design and conduct of the study; the collection, management, analysis, and interpretation of the data; the preparation, review, or approval of the manuscript; of the decision to submit the manuscript for publication.

## PATIENT CONSENT STATEMENT

The patients described in this case series have completed and signed a consent form for publication. The original signed forms are held by the institution.

## Data Availability

The data that support the findings of this study are available from the corresponding author upon reasonable request.
